# Establishment of an innovative and sustainable PCR technique for 1534 locus mutation of the knockdown resistance (*kdr*) gene in the dengue vector *Aedes albopictus*

**DOI:** 10.1186/s13071-019-3829-5

**Published:** 2019-12-26

**Authors:** Cai-Ying Zhu, Chun-Chun Zhao, Yi-Guan Wang, De-Ling Ma, Xiu-Ping Song, Jun Wang, Feng-Xia Meng

**Affiliations:** 1 State Key Laboratory of Infectious Disease Prevention and Control, National Institute for Communicable Disease Control and Prevention, Chinese Center for Disease Control and Prevention, Collaborative Innovation Center for Diagnosis and Treatment of Infectious Diseases, WHO Collaborating Center for Vector Surveillance and Management, Beijing, 102206 China; 2 School of Biological Sciences, University of Queensland, St Lucia, QLD 4072 Australia; 3 Eco-Global Pest Solutions Australia/Termite Doctor Pty Ltd, Archerfield, QLD 4108 Australia

**Keywords:** *Aedes albopictus*, *kdr*, Mutation, Allele-specific PCR, Insecticide resistance

## Abstract

**Background:**

Mutation of the voltage-gated sodium channel (VGSC) gene, or knockdown resistance (*kdr*) gene, is an important resistance mechanism against DDT and pyrethroids for dengue vector *Aedes albopictus*. A phenylalanine to serine (F1534S), leucine (F1534L) and cysteine (F1534C) substitution were detected in many *Ae. albopictus* populations around the world, and the mutant allele frequencies have been increasing in recent years. Therefore, it is essential to establish a simple, time-saving and cost-effective procedure to monitor the alleles in large-scale studies.

**Methods:**

Based on the mutation genotypes of the 1534 locus in the *kdr* gene, F/F, F/S, F/C, F/L, S/S, C/C, L/L and S/C, we designed specific forward and reverse primers and optimized the reaction conditions for establishing of the allele-specific PCR(AS-PCR) detection technique. DNA sequencing in this study was taken as the gold standard, and used to determine the accuracy of AS-PCR.

**Results:**

The designed AS-PCR technique showed high specificity for distinguishing the mutations at the 1534 locus, as the accuracy for F/F, F/S, F/C, F/L, S/S, C/C and S/C were 100%, 95.35%, 100%, 100%, 100%, 100% and 100%, respectively.

**Conclusions:**

The designed AS-PCR technique effectively distinguished individual genotypes for the mutations at the 1534 locus in the *kdr* gene, which could facilitate the knockdown resistance surveillance in *Ae. albopictus* in large-scale studies
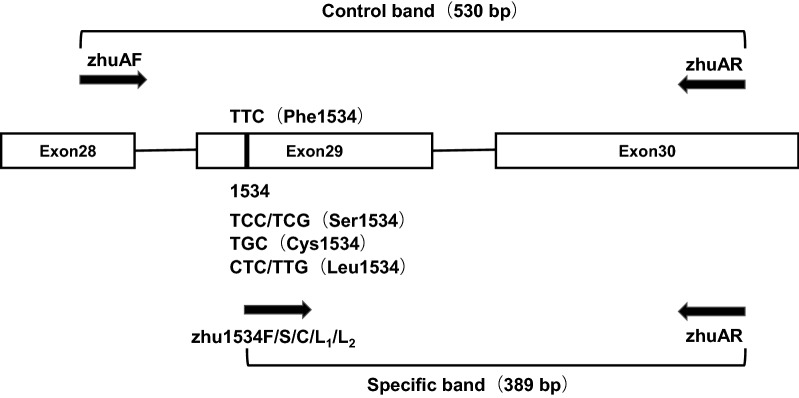
.

## Background

*Aedes albopictus*, the Asian tiger mosquito, is an important vector of dengue, chikungunya, yellow fever and Zika viruses and has emerged as a global public health threat [1, 2]. According to the WHO estimates, about 2.5 billion people are threatened by dengue fever worldwide, and more than 100 million people are infected with dengue virus every year [3]. Due to the high variability of the dengue virus, it is difficult to develop specific antiviral therapies and vaccines. Therefore, vector management is the sole method available for reducing the morbidity of *Aedes*-induced diseases and protecting public health [1].

The control of adult mosquitoes depends largely on the use of insecticides, such as carbamates, organophosphates and pyrethroids [4]. In particular, pyrethroids have been extensively and continuously used due to their low toxicity, broad-spectrum applications and high efficiency [5], which in turn has resulted in different levels of resistance to pyrethroids, i.e. deltamethrin [6, 7], permethrin [8, 9] and cyfluthrin [10]. Insecticide resistance surveillance plays an irreplaceable role in insecticide resistance management. With the development of molecular biology, the technique of insecticide resistance surveillance has gradually evolved from biological determination reflecting resistance phenotypes to molecular biological detection of resistance genotypes. Studies have shown that there is a mutation of the *kdr* gene in *Ae. albopictus*, which leads to a change in the corresponding amino acid, and the change is responsible for the molecular mechanism of resistance to DDT and pyrethroid insecticides [11]. According to current publications, the mutation of the *kdr* gene at the 1534 locus is highly complex and diverse [6, 12, 13]. There are six alleles at the 1534 locus, namely, wild-type TTC/F encoding phenylalanine, mutant TCC/S and TCG/S encoding serine, mutant TGC/C encoding cysteine, and CTC/L and TTG/L encoding leucine. In addition, there are eight genotypes in the 1534 locus, namely, wild homozygous F/F, wild/mutant heterozygotes F/S, F/C and F/L, mutant homozygous S/S, C/C and L/L as well as mutant heterozygous S/C [1, 14]. Classically, the *kdr* mutation in *Ae. albopictus* has been determined by nucleotide sequencing. Although direct sequencing is a gold standard and considered to be the most accurate method due to its high accuracy, it is expensive, time-consuming and inapplicable to large sample sizes. A simple and cost-effective method for single nucleotide polymorphism (SNP) genotyping would increase the accessibility of an average research laboratory to SNP markers. This allows SNP to be examined in laboratories with minimal equipment and in large-scale [15]. AS-PCR is such a candidate method that has already been applied to *Ae. aegypti*, *Culex quinquefasciatus*, *Culex pipiens pallens* and *Anopheles sinensis* [16–19]. Based on the principle of AS-PCR, a common forward primer and several forward primers are designed for each allele, with their 3ʹ-ends matched to wild and mutant alleles, respectively, and all the forward primers shared a common reverse primer. As the primer extension in the process of PCR starts from the 3ʹ-end, if the base of 3ʹ-end of the primer complements the template, the PCR could proceed successfully, thus obtaining a band with specific length in electrophoresis. Alternatively, if the primer doesn’t complementary to the template, then no specific band can be obtained [20–22]. This assay aims to develop an AS-PCR detection technique for each allele of the 1534 locus in the *kdr* gene in *Ae. albopictus*, which could reduce the cost and time of monitoring the mutant allele frequencies.

## Methods

### Collection of mosquitoes

A total of 255 *Ae. albopictus* adults were collected by backpack electric mosquito suction device in 2018 from Guizhou, Fujian, Guangdong, Hainan, Yunnan and Jiangsu provinces in China. In addition, we also studied some other field population reared in our laboratory, which were collected from Shanghai, Zhejiang, Jiangxi, Beijing, Guangdong and Hainan in China, between 2009 and 2010. Detailed information on *Ae. albopictus* populations is listed in Table 1.

**Table 1:** List of *Ae. albopictus* populations used for AS-PCR detection technique studies

Collection site	Collection date	Longitude (E)	Latitude (N)	*N*^a^	*n*^b^	Genotype^c^							
						FF	FS	SS	FC	CC	SC	FL	LL
Xingyi, Guizhou	September 2018	104.89	25.10	32	32	1	13	18	0	0	0	0	0
Jinjiang, Fujian	July 2018	118.58	24.82	16	16	6	9	1	0	0	0	0	0
Taijiang, Fujian	April 2018	119.31	26.07	32	20	4	7	0	4	0	5	0	0
Minhou, Fujian	April 2018	119.37	25.91	16	16	2	7	4	2	0	1	0	0
Fuqing, Fujian	April 2018	119.41	25.73	16	16	8	7	1	0	0	0	0	0
Baiyunshan, Guangdong	July 2018	113.30	23.18	16	5	0	0	0	0	0	2	3	0
Baiyun, Guangdong	July 2018	113.27	23.19	16	2	0	0	0	0	0	0	2	0
Huangpu, Guangdong	July 2018	113.45	23.11	32	2	0	0	0	0	0	0	1	1
Yongxing, Hainan	July 2018	110.26	19.89	13	2	0	0	0	0	0	1	1	0
Xiuzhong, Hainan	July 2018	110.27	20.01	14	4	0	0	0	0	0	4	0	0
Jinghong, Yunnan	September 2018	100.81	21.99	20	1	0	0	0	0	0	0	1	0
Nanjing, Jiangsu	June 2018	118.78	32.04	32	11	9	0	2	0	0	0	0	0
Zhabei, Shanghai	September 2009	121.27	31.15	16	8	0	0	0	4	4	0	0	0
Cixi, Zhejiang	July 2010	121.14	30.10	16	6	0	0	0	4	2	0	0	0
Nanchang, Jiangxi	August 2010	115.89	28.68	16	4	0	0	0	3	1	0	0	0
Changping, Beijing	September 2009	116.15	40.10	16	7	0	0	0	5	2	0	0	0
Guangzhou, Guangdong	January 2010	113.15	23.06	16	10	0	0	0	6	4	0	0	0
Haikou, Hainan	November 2009	110.20	20.01	16	3	0	0	0	3	0	0	0	0

^a^Samples of *Ae. albopictus* collected in 2018 and populations genotyped that collected between 2009 and 2010

^b^Samples used for AS-PCR detection technique studies

^c^Samples of each genotype used for AS-PCR detection technique studies

### Genotyping

Genomic DNA from each alcohol-preserved mosquito was extracted using Micro Tissue Genomic DNA Extraction Kit (BioTeke, Wuxi, China) and DNA/RNA Extractor-32 system (BioTeke). The primers aegSCF7 and aegSCR7 were utilized to amplify the domain III of the *kdr* gene, and the primer aegSCR8 was utilized to sequence in AUGCT Co. Ltd (Beijing, China) and TsingKe Co. Ltd (Beijing, China) [6] in the reverse direction. The sequences were analyzed using the software MEGA 7.0 and DNAstar 8.0. Finally, all individuals were genotyped (unpublished data) and 165 of them covering all eight genotypes were used for AS-PCR detection technique studies, including 30 wild homozygotes, 82 wild/mutant heterozygotes, 13 mutant heterozygotes and 40 mutant homozygotes.

### AS-PCR primer design

**Fig. 1:**
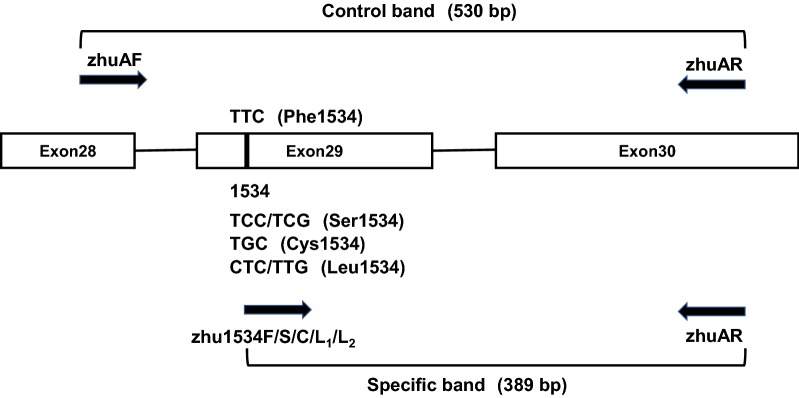
Schematic representation of the AS-PCR assay design with primer locations and predicted size of the PCR products

According to the partial voltage-gated sodium channel gene sequence of *Ae. albopictus* (GenBank: KC152046.1), the primer 5.0 was used to design the forward and reverse primers for AS-PCR amplification. The primer sequences are shown in Table 2. Among them, zhuAF and zhuAR are the common primers to amplify a control fragment with a length of 530 bp; zhu1534F, zhu1534S, zhu1534C, and zhu1534L_1_ and zhu1534L_2_ were designed for every mutant allele, and the size of the fragments amplified by the combination of the specific forward and common reverse primers was 389 bp (Fig. 1).

**Table 2:** The designed primers used for AS-PCR to determine the mutations at 1534 locus in *kdr* gene of *Ae. albopictus*

Primers	Orientation	Sequence (5ʹ–3ʹ)	Fragment size (bp)
zhuAF	Forward	ACTCGCGGGAGGTAAGTT	530
zhuAR	Reverse	GTCCGTCTGCTTGTAGTGAT	530
zhu1534F	Forward	CTTCGTGTTCTTCATCATCTT	389
zhu1534S	Forward	CTTCGTGTTCTTCATCATCTC	389
zhu1534C	Forward	CTTCGTGTTCTTCATCATCTG	389
zhu1534L_1_	Forward	CTTCGTGTTCTTCATCATCTTG	389
zhu1534L_2_	Forward	CTTCGTGTTCTTCATCATCC	389

### AS-PCR amplification reaction

The reaction was set up with four parallel reaction tubes for every DNA sample, i.e. tube F, S, C and L. The primers added into tube F were zhuAF, zhuAR and zhu1534F, which can be used to detect the TTC allele; the primers added into tube S were zhuAF, zhuAR and zhu1534S, which can be used to detect TCC and TCG alleles; the primers added into tube C were zhuAF, zhuAR and zhu1534C, which were used to detect the TGC allele; the primer combinations in tube L were zhuAF, zhuAR, zhu1534L_1_ and zhu1534L_2_, which were used to detect TTG and CTC alleles. For AS-PCR amplification reaction, each PCR was carried out in a 25 μl reaction volume, containing 12.5 μl of 2× Taq PCR premix (TransGen Biotech Co. Ltd., Beijing, China), 0.5 μl (10 mmol/l) common forward and reverse primers, 1 μl (10 mmol/l) specific forward primer, 2 μl of DNA template, and made up to 25 μl with ddH_2_O. The amplification consisted of 94 °C for 3 min pre-denaturation step, followed by 35 cycles of 94 °C for 30 s, 60 °C for 30 s and 72 °C for 30 s, and a final extension step at 72 °C for 5 min. The amplified PCR products were stored at 4 °C.

### Agarose gel electrophoresis

After PCR, the amplified products were subjected to 1.5% agarose gel with a 100 bp DNA ladder (TransGen Biotech Co. Ltd.) to estimate the size of the bands. The electrophoresis was run for 45 min at 120 V, 130 mA in TBE buffer and the gel was visualized using Gel Doc XR (Bio-Rad, California, USA).

### Statistical analysis

The results were statistically analyzed by Excel 2016 software. The data were subjected to Kappa consistency test and McNemar-Bowker test using SPSS 24.0 software.

## Results

### Electrophoresis

**Fig. 2:**
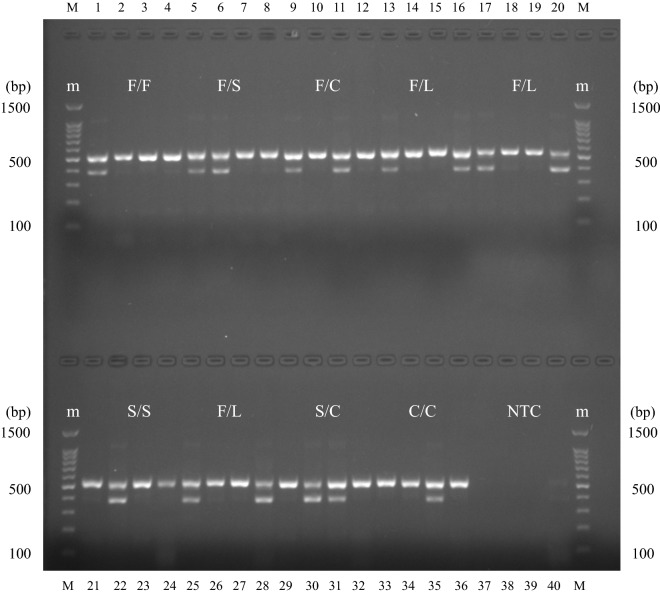
Gel Electrophoresis results of each mutant type amplified by AS-PCR. All panels represent each of the nine existing genotypes. Lane m: contains 100 bp DNA ladder. Lanes 1–36: contains PCR products by using 9 different mosquito DNA sample as template, in turn, F/F, F/S, F/C, F/L(CTC/L), F/L(TTG/L), S/S, L/L(TTG/L), S/C and C/C genotype. Lanes 1–4: F/F; Lanes 5–8: F/S; Lanes 9–12: F/C; Lanes 13–16: F/L; Lanes 17–20: F/L; Lanes 21–24: S/S; Lanes 25–28: F/L; Lanes 29–32: S/C; Lanes 33–36: C/C; Lanes 37–40: negative control in which distilled water was used as the template in the PCR reaction

A total of 165 samples covering the eight genotypes (see consensus sequences in Additional file 1) were tested, and all of them were successfully amplified by the AS-PCR. A total of 9 electrophoresis band patterns for each genotype were found, as shown in Fig. 2. The bands between 500 bp and 600 bp represented the positive control, and these around 400 bp corresponded to the specific wild/mutant allele fragments. The length of all bands obtained were in line with the expectations: Lanes 1–4 in Fig. 2 were for wild-type F/F; Lanes 5–8 were for wild/mutant heterozygote F/S; Lanes 9–12 were for wild/mutant heterozygote F/C; Lanes 13–16 were for wild/mutant heterozygote F/L (CTC/L); Lanes 17–20 were for wild/mutant heterozygote F/L (TTG/L); and Lanes 21–24 were for mutant homozygote S/S. Lanes 25–28 were for mutant homozygote L/L (TTG/L), which is non-specifically determined to be a wild/mutant heterozygote F/L (TTG/L) in this study. Lanes 29–32 were for mutant heterozygote S/C, Lanes 33–36 were for mutant homozygote C/C, and Lanes 37–40 were for negative controls for tubes F, S, C and L, respectively.

### Comparison of DNA sequencing with the AS-PCR

#### Mutant genotypes and frequencies

**Fig. 3:**
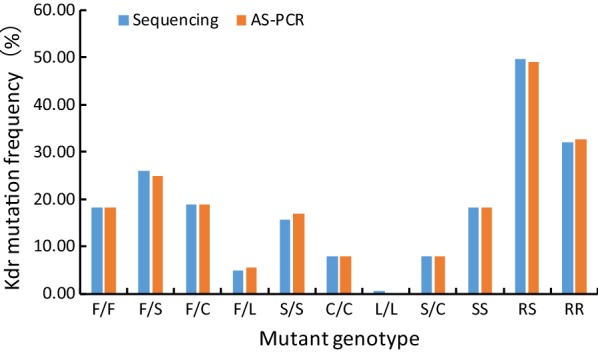
The sensitive/mutant genotypes and their frequency at the 1534 locus of the *kdr* gene in *Ae. albopictus*. F/F, F/S, F/C, F/L, S/S, C/C, L/L, S/C, before and after the “/” are the amino acids corresponding to the two alleles of the individual. *Abbreviations*: F, phenylalanine; S, serine; C; cysteine; L, leucine; SS, susceptible homozygote; RS, wild/mutant heterozygote; RR, mutant heterozygote and homozygote

In this study, sequencing was considered as the gold standard to determine genotypes. Sequencing results revealed 30 F/F genotypes, 31 F/C samples, 8 F/L samples, 26 S/S samples, 13 C/C samples and 13 S/C samples, which were completely consistent with the results determined by AS-PCR. This indicated that the AS-PCR established in this study was of 100% accuracy for the F/F, F/C, F/L, S/S, C/C and S/C mutations. However, there were slight discrepancies between the AS-PCR results and those from sequencing of other mutant genotypes. The results of AS-PCR showed that of the 43 F/S individuals determined by sequencing, 41 were F/S, and 2 were misdiagnosed as S/S, resulting in an accuracy of 95.35%. In addition, 1 L/L (TTG/TTG) individual detected by sequencing was identified as F/L by AS-PCR, as shown in Table 3 and Fig. 3. Kappa consistency test was performed on the test results of the two methods, and the results suggest that the agreement in rating of genotypes identification of the two methods was not due to chance (*P* < 0.0001). The Kappa value was 0.978, indicating an almost perfect agreement of the test results by the two methods. In terms of genotypes, 165 nucleotide sequences were amplified by AS-PCR and electrophoresis was performed, among which 30 (18.81%) were susceptible homozygotes, 81 (49.09%) were wild/mutant heterozygotes, 13 (7.88%) were mutant heterozygotes, and 41 (24.85%) were mutant homozygotes. Sequencing results of the above 165 *Ae. albopictus* showed that 30 (18.18%) were susceptible homozygotes, 82 (49.70%) were wild/mutant heterozygotes. Mutant heterozygotes were identified in 13 (7.88%) samples, and 40 (24.24%) were mutant homozygotes (see Fig. 3). The McNemar-Bowker test results showed no significant difference in the levels of resistance genotypes detected by the two methods (*χ*^2^ = 0.333, *P* = 0.564).

**Table 3:** The genotypes and frequencies of *Ae. albopictus kdr* gene at the 1534 locus

Genotypes	F/F *n* (%)	F/S *n* (%)	F/C *n* (%)	F/L *n* (%)	S/S *n* (%)	C/C *n* (%)	L/L *n* (%)	S/C *n* (%)
Sequencing	30 (18.18)	43 (26.06)	31 (18.79)	8 (4.85)	26 (15.76)	13 (7.88)	1 (0.61)	13 (7.88)
AS-PCR	30 (18.18)	41 (24.85)	31 (18.79)	9 (5.45)	28 (16.97)	13 (7.88)	0 (0.00)	13 (7.88)

*Note*: F/F, F/S, F/C, F/L, S/S, C/C, L/L, S/C, before and after the “/” line are the amino acids corresponding to the two alleles of the individual

*Abbreviations*: F, phenylalanine; S, serine; C, cysteine; L, leucine, n, number of individuals

#### Alleles and frequencies

**Fig. 4:**
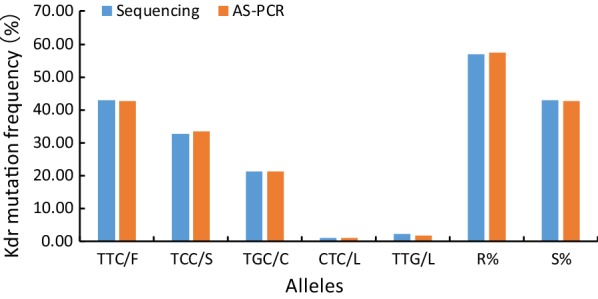
The sensitive/mutant alleles and their frequency at the 1534 locus of the *kdr* gene in *Ae. albopictus*. TTC, TCC, TGC, CTC, TTG are codons. *Abbreviations*: F, phenylalanine; S, serine; C, cysteine; L, leucine; R%, resistance allele frequency; S%, susceptible allele frequency

In terms of alleles, 165 nucleic acid samples were tested and 5 alleles were obtained, namely wild type TTC/F, mutant TCC/S, TGC/C, CTC/L and TTG/L as shown in Table 4 and Fig. 4. The Kappa consistency test resulted in Kappa = 0.986 (*P* < 0.0001), suggesting the results of the allele frequencies detected by the two methods were of almost perfect agreement. The results of the two techniques were also subjected to McNemar-Bowker test, showing no statistically significant difference in the frequency of alleles between the two methods (*χ*^2^ = 3.000, *P* = 0.223).

**Table 4:** The alleles and frequencies of *Ae. albopictus kdr* gene at the 1534 locus

Alleles	TTC/F *n* (%)	TCC/S *n* (%)	TGC/C *n* (%)	CTC/L *n* (%)	TTG/L *n* (%)
Sequencing	142 (43.03)	108 (32.73)	70 (21.21)	3 (0.91)	7 (2.12)
AS-PCR	141 (42.73)	110 (33.33)	70 (21.21)	3 (0.91)	6 (1.81)

*Note*: TTC/F, TCC/S, TGC/C, CTC/L, TTG/L, before the “/” is the codon; after the “/” is the corresponding amino acid

*Abbreviations*: F, phenylalanine; S, serine; C, cysteine; L, leucine, n, number of individuals

From the perspective of resistance level, 165 DNA templates were amplified by AS-PCR, and the resistance allele frequency detected was 57.27%, slightly higher than that detected by sequencing (56.97%), and the susceptible allele frequency of 42.73% examined by AS-PCR was slightly lower than that detected by sequencing (43.03%), as shown in Table 5 and Fig. 4.

**Table 5:** The genotypes and frequencies of *Ae. albopictus kdr* gene at the 1534 locus

Methods	Sample size	Genotype			Genotype frequency			Allele frequency	
		SS	RS	RR	SS%	RS%	RR%	S%	R%
Sequencing	165	30	82	53	18.18	49.70	32.12	43.03	56.97
AS-PCR	165	30	81	54	18.18	49.09	32.73	42.73	57.27

*Abbreviations*: SS, susceptible homozygote; SS%, SS/(SS + RS + RR) × 100%; RS, wild/mutant heterozygote; RS%, RS/(SS + RS + RR) × 100%; RR, mutant heterozygote and mutant homozygote; RR%, RR/(SS + RS + RR) × 100%; S%, SS% + 0.5 × RS%; R%, 1 − S%

## Discussion

Since no specific treatment and/or vaccines are commonly available against chikungunya and Zika, and since the vaccine for dengue has been deployed only very recently in a small number of experimental areas [23], the control of vectors and personal protection against mosquitos *via* chemical insecticides remain the only available tools for preventing and controlling these arboviral diseases [24, 25]. However, the large-scale deployment of insecticides to control mosquitoes has allowed the generation and dramatical development of resistance worldwide [26]. At present, insecticide resistance surveillance in China primarily relies on larval and adult bioassays, which is not only a labor-intensive multi-step process, but also has a high standard for mosquito samples for testing. Moreover, only when the resistance gene develops to the resistance homozygote in the population, can it be detected by the larval and adult bioassays, so these could not play a monitoring role at the early stage of insecticide resistance occurrence. However, the early detection of resistance alleles is essential for the successful implementation of insecticide resistance management strategies, and some molecular biological approaches such as direct sequencing and AS-PCR can achieve this [27]. The development of molecular biology has enabled us to detect resistance-related genes and mutations on a molecular level. In the present study, we successfully established an AS-PCR detection technique to test mutations of *Ae. albopictus kdr* gene at the 1534 locus. The results of the AS-PCR were in an almost perfect agreement with those of DNA sequencing. AS-PCR has several advantages to determine resistance levels. First, compared with the classical biological assays, AS-PCR does not have specific requirements for mosquito samples. Secondly, AS-PCR is able to cut the cost of DNA sequencing for determining individual genotypes. Although there were slight discrepancies between the AS-PCR results and those from DNA sequencing, these did not affect the overall detection results. A similar situation was also found in the previous studies [28, 29]. Therefore, AS-PCR is suitable for the rapid determining of the *kdr* mutation in *Ae. albopictus* field populations, and is of high practical value when *kdr* mutation detection needs to be implemented on a large scale. Considering the complexity and diversity of mutations at the 1534 locus of *Ae. albopictus kdr* gene, it is recommended to select the corresponding reaction tube in combination with the mutation characteristics of each region.

Of course, the AS-PCR detection technique established in this study also has its own drawbacks. First, the mutant allele of leucine in this study includes two types: CTC/L and TTG/L. While the former allele can be accurately detected by AS-PCR, the latter cannot. Since the primer added to the F tube ends with base TT, both the allele TTG/L and TTC/F can be amplified. Therefore, when the sample is TTG/L homozygous, specific bands can also be amplified in the F tube, leading to the determination of TTG/L homozygote as F/L (TTC/TTG) heterozygote. However, we tried to modify the primer zhuAF to end with TTC, and the results revealed that the other mutation types showed more non-specific bands, which greatly affected the reliability and accuracy of the AS-PCR. Considering that L/L genotype is rarely detected in wild populations, the effect of this misdiagnosis may be negligible. Secondly, genotype identification accuracy of mutant heterozygous F/S (TTC/TCC) samples was 95.35%, due to 2 out of 43 showing as homozygous S/S by AS-PCR, but heterozygous F/S by direct sequencing; a similar situation was found in other studies [28, 29]. Theoretically, there are 10 possible genotypes at the 1534 locus of the *kdr* gene in *Ae. albopictus*, i.e. F/F, S/S, C/C, L/L, F/S, F/C, F/L, S/C, S/L and C/L (mutant heterozygotes S/L and C/L have not yet been reported). Actually, the mutant alleles at the 1534 locus are further complicated by the fact that the same amino acid can be encoded by several codons. For example, serine can be encoded by both the codon TCC and TCG. All these considerations make the mutations at this locus complicated and diverse, and limit the accessibility to all mutant genotypes. Thirdly, a limitation of this current study was the small size of the number of some resistant mutations, especially the mutations L/L and F/L. The reason was that no more individuals with these resistant mutations were obtained in this experiment. Finally, the AS-PCR tool established in this study requires four lanes for each sample. Consequently, more cost-effective and rapid single-tube assays, e.g. multiplex PCR and tri-allelic PCR [18, 29], should be taken into account urgently. In general, the degree of resistance and the frequency of susceptible alleles detected by AS-PCR were almost the same as those by sequencing, meaning AS-PCR can accurately reflect the resistance levels of a given *Ae. albopictus* population at the molecular level. Although not all mutant genotypes at the 1534 locus were detected in this study, we have taken all mutations into consideration in the primer design stage.

## Conclusions

The AS-PCR developed in this study has high accuracy to detect *kdr* mutations in the 1534 locus of the *kdr* gene in *Ae. albopictus*, which allows identification of all possible genotypes without DNA sequencing. The method proved to be highly reliable and would benefit future studies in determining the extent of *kdr* mutations in *Ae. albopictus* populations and could assist decision-making for resistance management.

## Supplementary information

**Additional file 1: Alignment S1.** ClustalW alignment for eight genotypes in the 1534 locus of the *kdr* gene in *Ae. albopictus*. The underlined uppercase letters referring to the codons in the 1534 locus, and the letter highlighted in yellow corresponds to the insertion. All the alignments originate from the sequences sequenced in reverse direction by the primer aegSCR8.

## Data Availability

Data supporting the conclusions of this article are provided within the article and its additional file. All the sequences were submitted to the GenBank database under the accession numbers MN433712-MN433876.

## Abbreviations

AS-PCR: allele-specific PCR
DDT: dichlorodiphenyltrichloroethane
VGSC: voltage-gated sodium channel
*kdr*: knockdown resistance
F1534S or F/S: phenylalanine to serine substitution at position 1534
F1534L or F/L: phenylalanine to leucine substitution at position 1534
F1534C or F/C: phenylalanine to cysteine substitution at position 1534
F/F: phenylalanine allele at position 1534
S/S: serine allele at position 1534
C/C: cysteine allele at position 1534
L/L: leucine allele at position 1534
S/C: serine allele at position 1534
TTC/F: codon TTC encoding phenylalanine
TCC/S: codon TCC encoding serine
TCG/S: codon TCG encoding serine
TGC/C: codon TGC encoding cysteine
CTC/L: codon CTC encoding leucine
TTG/L: codon TTG encoding leucine
SNP: single nucleotide polymorphism
TBE: tris-borate-EDTA buffer

## References

[1] Li Y, Xu J, Zhong D, Zhang H, Yang W, Zhou G (2018). Evidence for multiple-insecticide resistance in urban *Aedes albopictus* populations in southern China. Parasites Vectors..

[2] Jupille H, Seixas G, Mousson L, Sousa CA, Failloux A (2016). Zika virus, a new threat for Europe?. PLoS Negl Trop Dis..

[3] Kraemer M, Sinka M, Duda K, Mylne A, Shearer F, Barker C (2015). The global distribution of the arbovirus vectors *Aedes aegypti* and *Ae. albopictus*. ELife..

[4] Fotakis EA, Chaskopoulou A, Grigoraki L, Tsiamantas A, Kounadi S, Georgiou L (2017). Analysis of population structure and insecticide resistance in mosquitoes of the genus *Culex*, *Anopheles* and *Aedes* from different environments of Greece with a history of mosquito borne disease transmission. Acta Trop..

[5] Moyes CL, Vontas J, Martins AJ, Ng LC, Koou SY, Dusfour I (2017). Contemporary status of insecticide resistance in the major *Aedes* vectors of arboviruses infecting humans. PLoS Negl Trop Dis..

[6] Kasai S, Ng LC, Lam-Phua SG, Tang CS, Itokawa K, Komagata O (2011). First detection of a putative knockdown resistance gene in major mosquito vector, *Aedes albopictus*. Jpn J Infect Dis..

[7] Kushwah R, Mallick P, Ravikumar H, Dev V, Kapoor N, Adak T (2015). Status of DDT and pyrethroid resistance in Indian *Aedes albopictus* and absence of knockdown resistance (*kdr*) mutation. J Vector Borne Dis..

[8] Chuaycharoensuk T, Juntarajumnong W, Boonyuan W, Bangs MJ, Akratanakul P, Thammapalo S (2011). Frequency of pyrethroid resistance in *Aedes aegypti* and *Aedes albopictus* (Diptera: Culicidae) in Thailand. J Vector Ecol..

[9] Ishak IH, Jaal Z, Ranson H, Wondji CS (2015). Contrasting patterns of insecticide resistance and knockdown resistance (*kdr*) in the dengue vectors *Aedes aegypti* and *Aedes albopictus* from Malaysia. Parasites Vectors..

[10] Rath A, Mohanty I, Hazra RK (2018). Insecticide susceptibility status of invasive *Aedes albopictus* across dengue endemic districts of Odisha, India. Pest Manag Sci..

[11] Auteri M, La Russa F, Blanda V, Torina A. Insecticide resistance associated with *kdr* mutations in *Aedes albopictus*: an update on worldwide evidences. Biomed Res Int. 2018:3098575. 10.1155/2018/3098575 PMC6098900 30175124

[12] Xu J, Bonizzoni M, Zhong D, Zhou G, Cai S, Li Y (2016). Multi-country survey revealed prevalent and novel F1534S mutation in voltage-gated sodium channel (VGSC) gene in *Aedes albopictus*. PLoS Negl Trop Dis..

[13] Marcombe S, Farajollahi A, Healy S, Clark G, Fonseca D (2014). Insecticide resistance status of United States populations of *Aedes albopictus* and mechanisms involved. PLoS ONE..

[14] Aguirre-Obando OA, Martins AJ, Navarro-Silva MA (2017). First report of the Phe1534Cys *kdr* mutation in natural populations of *Aedes albopictus* from Brazil. Parasites Vectors..

[15] Bundock PC, Cross MJ, Shapter FM, Henry RJ (2006). Robust allele-specific polymerase chain reaction markers developed for single nucleotide polymorphisms in expressed barley sequences. Theor Appl Genet..

[16] Li CX, Kaufman PE, Xue RD, Zhao MH, Wang G, Yan T (2015). Relationship between insecticide resistance and *kdr* mutations in the dengue vector *Aedes aegypti* in southern China. Parasites Vectors..

[17] Stenhouse SA, Plernsub S, Yanola J, Lumjuan N, Dantrakool A, Choochote W (2013). Detection of the V1016G mutation in the voltage-gated sodium channel gene of *Aedes aegypti* (Diptera: Culicidae) by allele-specific PCR assay, and its distribution and effect on deltamethrin resistance in Thailand. Parasites Vectors..

[18] Silva Martins WF, Silva Pereira BN, Vieira Alves AT, Murphy A, Silva Martins PG, Weetman D (2019). Development and application of a tri-allelic PCR assay for screening Vgsc-L1014F *kdr* mutations associated with pyrethroid and organochlorine resistance in the mosquito *Culex quinquefasciatus*. Parasites Vectors..

[19] Liu H, Cheng P, Huang X, Dai Y, Wang H, Liu L (2013). Identification of TCT, a novel knockdown resistance allele mutation and analysis of resistance detection methods in the voltage-gated Na^+^ channel of *Culex pipiens pallens* from Shandong Province, China. Mol Med Rep..

[20] Kim MY, Van K, Lestari P, Moon JK, Lee SH (2005). SNP identification and SNAP marker development for a *GmNARK* gene controlling supernodulation in soybean. Theor Appl Genet..

[21] Gaudet M, Fara AG, Beritognolo I, Sabatti M (2009). Allele-specific PCR in SNP genotyping. Methods Mol Biol..

[22] Yang P, Song Y, Xia X, Zhang A (2019). Rapid screening mutations of first-line-drug-resistant genes in *Mycobacterium tuberculosis* strains by allele-specific real-time quantitative PCR. PeerJ..

[23] Gottschamel J, Lössl A, Ruf S, Wang Y, Skaugen M, Bock R (2016). Production of dengue virus envelope protein domain III-based antigens in tobacco chloroplasts using inducible and constitutive expression systems. Plant Mol Biol..

[24] Weaver SC, Costa F, Garcia-Blanco MA, Ko AI, Ribeiro GS, Saade G (2016). Zika virus: history, emergence, biology, and prospects for control. Antivir Res..

[25] Debboun M, Strickman D (2013). Insect repellents and associated personal protection for a reduction in human disease. Med Vet Entomol..

[26] Faucon F, Dusfour I, Gaude T, Navratil V, Boyer F, Chandre F (2015). Identifying genomic changes associated with insecticide resistance in the dengue mosquito *Aedes aegypti* by deep targeted sequencing. Genome Res..

[27] Muthusamy R, Shivakumar MS (2015). Susceptibility status of *Aedes aegypti* (L.) (Diptera: Culicidae) to temephos from three districts of Tamil Nadu, India. J Vector Borne Dis..

[28] Yanola J, Somboon P, Walton C, Nachaiwieng W, Somwang P, Prapanthadara L (2011). High-throughput assays for detection of the F1534C mutation in the voltage-gated sodium channel gene in permethrin-resistant *Aedes aegypti* and the distribution of this mutation throughout Thailand. Trop Med Int Health..

[29] Saingamsook J, Saeung A, Yanola J, Lumjuan N, Walton C, Somboon P (2017). A multiplex PCR for detection of knockdown resistance mutations, V1016G and F1534C, in pyrethroid-resistant *Aedes aegypti*. Parasites Vectors..

