# Supplementary material for: Establishment of an innovative and sustainable PCR technique for 1534 locus mutation of the knockdown resistance (kdr) gene in the dengue vector Aedes albopictus
Source: Parasit Vectors. 2019 Dec 26;12:603. doi: 10.1186/s13071-019-3829-5 (PMC6933705; doi:10.1186/s13071-019-3829-5)
Supplement: Supplementary file 1 — Additional file 1: Alignment S1. ClustalW alignment for eight genotypes in the 1534 locus of the kdr gene in Ae. albopictus. The underlined uppercase letters referring to the codons in the 1534 locus, and the letter highlighted in yellow corresponds to the insertion. All the alignments originate from the sequences sequenced in reverse direction by the primer aegSCR8. [file 13071_2019_3829_MOESM1_ESM.docx]

**Additional file 1**

**F1534F**

KC152046.1 ATTCGCGAGACCAACATCTACATGTACCTCTACTTCGTGTTCTTCATCATCTTCGGGTCGTTCTTCACCCTCAACCTGTTCATCGGTGTC

#SS **S1534S**

FJ-11 ATTCGCGAGACCAACATCTACATGTACCTCTACTTCGTGTTCTTCATCATCTCCGGGTCGTTCTTCACCCTTAATCTGTTCATCGGTGTC

FJ-34 ATTCGCGAGACCAACATCTACATGTACCTCTACTTCGTGTTCTTCATCATCTCCGGGTCGTTCTTCACCCTTAATCTGTTCATCGGTGTC

FJ-39 ATTCGCGAGACCAACATCTACATGTACCTCTACTTCGTGTTCTTCATCATCTCCGGGTCGTTCTTCACCCTTAATCTGTTCATCGGTGTC

FJ-45 ATTCGCGAGACCAACATCTACATGTACCTCTACTTCGTGTTCTTCATCATCTCCGGGTCGTTCTTCACCCTTAATCTGTTCATCGGTGTC

FJ-47 ATTCGCGAGACCAACATCTACATGTACCTCTACTTCGTGTTCTTCATCATCTCCGGGTCGTTCTTCACCCTTAATCTGTTCATCGGTGTC

FJ-59 ATTCGCGAGACCAACATCTACATGTACCTCTACTTCGTGTTCTTCATCATCTCCGGGTCGTTCTTCACCCTTAATCTGTTCATCGGTGTC

GZ-2 ATTCGCGAGACCAACATCTACATGTACCTCTACTTCGTGTTCTTCATCATCTCCGGGTCGTTCTTCACCCTTAATCTGTTCATCGGTGTC

GZ-4 ATTCGCGAGACCAACATCTACATGTACCTCTACTTCGTGTTCTTCATCATCTCCGGGTCGTTCTTCACCCTTAATCTGTTCATCGGTGTC

GZ-5 ATTCGCGAGACCAACATCTACATGTACCTCTACTTCGTGTTCTTCATCATCTCCGGGTCGTTCTTCACCCTTAATCTGTTCATCGGTGTC

GZ-7 ATTCGCGAGACCAACATCTACATGTACCTCTACTTCGTGTTCTTCATCATCTCCGGGTCGTTCTTCACCCTTAATCTGTTCATCGGTGTC

GZ-8 ATTCGCGAGACCAACATCTACATGTACCTCTACTTCGTGTTCTTCATCATCTCCGGGTCGTTCTTCACCCTTAATCTGTTCATCGGTGTC

GZ-9 ATTCGCGAGACCAACATCTACATGTACCTCTACTTCGTGTTCTTCATCATCTCCGGGTCGTTCTTCACCCTTAATCTGTTCATCGGTGTC

GZ-11 ATTCGCGAGACCAACATCTACATGTACCTCTACTTCGTGTTCTTCATCATCTCCGGGTCGTTCTTCACCCTTAATCTGTTCATCGGTGTC

GZ-13 ATTCGCGAGACCAACATCTACATGTACCTCTACTTCGTGTTCTTCATCATCTCCGGGTCGTTCTTCACCCTTAATCTGTTCATCGGTGTC

GZ-14 ATTCGCGAGACCAACATCTACATGTACCTCTACTTCGTGTTCTTCATCATCTCCGGGTCGTTCTTCACCCTTAATCTGTTCATCGGTGTC

GZ-15 ATTCGCGAGACCAACATCTACATGTACCTCTACTTCGTGTTCTTCATCATCTCCGGGTCGTTCTTCACCCTTAATCTGTTCATCGGTGTC

GZ-17 ATTCGCGAGACCAACATCTACATGTACCTCTACTTCGTGTTCTTCATCATCTCCGGGTCGTTCTTCACCCTTAATCTGTTCATCGGTGTC

GZ-20 ATTCGCGAGACCAACATCTACATGTACCTCTACTTCGTGTTCTTCATCATCTCCGGGTCGTTCTTCACCCTTAATCTGTTCATCGGTGTC

GZ-21 ATTCGCGAGACCAACATCTACATGTACCTCTACTTCGTGTTCTTCATCATCTCCGGGTCGTTCTTCACCCTTAATCTGTTCATCGGTGTC

GZ-22 ATTCGCGAGACCAACATCTACATGTACCTCTACTTCGTGTTCTTCATCATCTCCGGGTCGTTCTTCACCCTTAATCTGTTCATCGGTGTC

GZ-26 ATTCGCGAGACCAACATCTACATGTACCTCTACTTCGTGTTCTTCATCATCTCCGGGTCGTTCTTCACCCTTAATCTGTTCATCGGTGTC

GZ-27 ATTCGCGAGACCAACATCTACATGTACCTCTACTTCGTGTTCTTCATCATCTCCGGGTCGTTCTTCACCCTTAATCTGTTCATCGGTGTC

GZ-29 ATTCGCGAGACCAACATCTACATGTACCTCTACTTCGTGTTCTTCATCATCTCCGGGTCGTTCTTCACCCTTAATCTGTTCATCGGTGTC

GZ-31 ATTCGCGAGACCAACATCTACATGTACCTCTACTTCGTGTTCTTCATCATCTCCGGGTCGTTCTTCACCCTTAATCTGTTCATCGGTGTC

NJ-9 ATTCGCGAGACCAACATCTACATGTACCTCTACTTCGTGTTCTTCATCATCTCCGGGTCGTTCTTCACCCTTAATCTGTTCATCGGTGTC

NJ-30 ATTCGCGAGACCAACATCTACATGTACCTCTACTTCGTGTTCTTCATCATCTCCGGGTCGTTCTTCACCCTTAATCTGTTCATCGGTGTC

#FC **F1534C**

CP-18 ATTCGCGAGACCAACATCTACATGTACCTCTACTTTGTGTTCTTCATCATCTTCGGGTCGTTCTTCACGCTGAATCTGTTCATCGGTGTC

CP-24 ATTCGCGAGACCAACATCTACATGTACCTCTACTTCGTGTTCTTCATCATCTGCGGGTCGTTCTTCACGCTGAATCTGTTCATCGGTGTC

CP-26 ATTCGCGAGACCAACATCTACATGTACCTCTACTTTGTGTTCTTCATCATCTTCGGGTCGTTCTTCACGCTGAATCTGTTCATCGGTGTC

CP-31 ATTCGCGAGACCAACATCTACATGTACCTCTACTTTGTGTTCTTCATCATCTTCGGGTCGTTCTTCACGCTGAATCTGTTCATCGGTGTC

CP-32 ATTCGCGAGACCAACATCTACATGTACCTCTACTTTGTGTTCTTCATCATCTTCGGGTCGTTCTTCACGCTGAATCTGTTCATCGGTGTC

CX-2 ATTCGCGAGACCAACATCTACATGTACCTCTACTTCGTGTTCTTCATCATCTTCGGGTCGTTCTTCACGCTGAATCTGTTCATCGGTGTC

CX-9 ATTCGCGAGACCAACATCTACATGTACCTCTACTTCGTGTTCTTCATCATCTTCGGGTCGTTCTTCACGCTGAATCTGTTCATCGGTGTC

CX-15 ATTCGCGAGACCAACATCTACATGTACCTCTACTTCGTGTTCTTCATCATCTGCGGGTCGTTCTTCACGCTGAATCTGTTCATCGGTGTC

CX-16 ATTCGCGAGACCAACATCTACATGTACCTCTACTTCGTGTTCTTCATCATCTTCGGGTCGTTCTTCACGCTGAATCTGTTCATCGGTGTC

FJ-20 ATTCGCGAGACCAACATCTACATGTACCTCTACTTTGTGTTCTTCATCATCTTCGGGTCGTTCTTCACCCTCAACCTGTTCATCGGTGTC

FJ-24 ATTCGCGAGACCAACATCTACATGTACCTCTACTTTGTGTTCTTCATCATCTTCGGGTCGTTCTTCACCCTCAACCTGTTCATCGGTGTC

FJ-30 ATTCGCGAGACCAACATCTACATGTACCTCTACTTCGTGTTCTTCATCATCTGCGGGTCGTTCTTCACCCTCAACCTGTTCATCGGTGTC

FJ-32 ATTCGCGAGACCAACATCTACATGTACCTCTACTTCGTGTTCTTCATCATCTGCGGGTCGTTCTTCACCCTCAACCTGTTCATCGGTGTC

FJ-33 ATTCGCGAGACCAACATCTACATGTACCTCTACTTCGTGTTCTTCATCATCTTCGGGTCGTTCTTCACTCTCAACCTGTTCATCGGTGTC

FJ-35 ATTCGCGAGACCAACATCTACATGTACCTCTACTTCGTGTTCTTCATCATCTTCGGGTCGTTCTTCACTCTCAACCTGTTCATCGGTGTC

GZ-1 ATTCGCGAGACCAACATCTACATGTACCTCTACTTTGTGTTCTTCATCATCTTCGGGTCGTTCTTCACGCTGAATCTGTTCATCGGTGTC

GZ-3 ATTCGCGAGACCAACATCTACATGTACCTCTACTTTGTGTTCTTCATCATCTTCGGGTCGTTCTTCACGCTGAATCTGTTCATCGGTGTC

GZ-6 ATTCGCGAGACCAACATCTACATGTACCTCTACTTTGTGTTCTTCATCATCTTCGGGTCGTTCTTCACGCTGAATCTGTTCATCGGTGTC

GZ-7 ATTCGCGAGACCAACATCTACATGTACCTCTACTTTGTGTTCTTCATCATCTTCGGGTCGTTCTTCACGCTGAATCTGTTCATCGGTGTC

GZ-10 ATTCGCGAGACCAACATCTACATGTACCTCTACTTTGTGTTCTTCATCATCTTCGGGTCGTTCTTCACGCTGAATCTGTTCATCGGTGTC

GZ-16 ATTCGCGAGACCAACATCTACATGTACCTCTACTTTGTGTTCTTCATCATCTTCGGGTCGTTCTTCACGCTGAATCTGTTCATCGGTGTC

HK-19 ATTCGCGAGACCAACATCTACATGTACCTCTACTTTGTGTTCTTCATCATCTTCGGGTCGTTCTTCACGCTGAATCTGTTCATCGGTGTC

HK-27 ATTCGCGAGACCAACATCTACATGTACCTCTACTTTGTGTTCTTCATCATCTTCGGGTCGTTCTTCACGCTGAATCTGTTCATCGGTGTC

HK-28 ATTCGCGAGACCAACATCTACATGTACCTCTACTTTGTGTTCTTCATCATCTTCGGGTCGTTCTTCACGCTGAATCTGTTCATCGGTGTC

NC-17 ATTCGCGAGACCAACATCTACATGTACCTCTACTTTGTGTTCTTCATCATCTTCGGGTCGTTCTTCACGCTGAATCTGTTCATCGGTGTC

NC-31 ATTCGCGAGACCAACATCTACATGTACCTCTACTTTGTGTTCTTCATCATCTGCGGGTCGTTCTTCACGCTGAATCTGTTCATCGGTGTC

NC-32 ATTCGCGAGACCAACATCTACATGTACCTCTACTTTGTGTTCTTCATCATCTTCGGGTCGTTCTTCACGCTGAATCTGTTCATCGGTGTC

SH-8 ATTCGCGAGACCAACATCTACATGTACCTCTACTTTGTGTTCTTCATCATCTTCGGGTCGTTCTTCACGCTGAATCTGTTCATCGGTGTC

SH-11 ATTCGCGAGACCAACATCTACATGTACCTCTACTTTGTGTTCTTCATCATCTGCGGGTCGTTCTTCACGCTGAATCTGTTCATCGGTGTC

SH-13 ATTCGCGAGACCAACATCTACATGTACCTCTACTTTGTGTTCTTCATCATCTGCGGGTCGTTCTTCACGCTGAATCTGTTCATCGGTGTC

SH-14 ATTCGCGAGACCAACATCTACATGTACCTCTACTTTGTGTTCTTCATCATCTTCGGGTCGTTCTTCACGCTGAATCTGTTCATCGGTGTC

#CC **C1534C**

CP-20 ATTCGCGAGACCAACATCTACATGTACCTCTACTTTGTGTTCTTCATCATCTGCGGGTCGTTCTTCACGCTGAATCTGTTCATCGGTGTC

CP-30 ATTCGCGAGACCAACATCTACATGTACCTCTACTTTGTGTTCTTCATCATCTGCGGGTCGTTCTTCACGCTGAATCTGTTCATCGGTGTC

CX-1 ATTCGCGAGACCAACATCTACATGTACCTCTACTTTGTGTTCTTCATCATCTGCGGGTCGTTCTTCACGCTGAATCTGTTCATCGGTGTC

CX-7 ATTCGCGAGACCAACATCTACATGTACCTCTACTTTGTGTTCTTCATCATCTGCGGGTCGTTCTTCACGCTGAATCTGTTCATCGGTGTC

GZ-11 ATTCGCGAGACCAACATCTACATGTACCTCTACTTTGTGTTCTTCATCATCTGCGGGTCGTTCTTCACGCTGAATCTGTTCATCGGTGTC

GZ-12 ATTCGCGAGACCAACATCTACATGTACCTCTACTTTGTGTTCTTCATCATCTGCGGGTCGTTCTTCACGCTGAATCTGTTCATCGGTGTC

GZ-14 ATTCGCGAGACCAACATCTACATGTACCTCTACTTTGTGTTCTTCATCATCTGCGGGTCGTTCTTCACGCTGAATCTGTTCATCGGTGTC

GZ-15 ATTCGCGAGACCAACATCTACATGTACCTCTACTTTGTGTTCTTCATCATCTGCGGGTCGTTCTTCACGCTGAATCTGTTCATCGGTGTC

NC-25 ATTCGCGAGACCAACATCTACTATGTACCTCTACTTTGTGTTCTTCATCATCTGCGGGTCGTTCTTCACGCTGAATCTGTTCATCGGTGTC

SH-5 ATTCGCGAGACCAACATCTACATGTACCTCTACTTTGTGTTCTTCATCATCTGCGGGTCGTTCTTCACGCTGAATCTGTTCATCGGTGTC

SH-10 ATTCGCGAGACCAACATCTACATGTACCTCTACTTTGTGTTCTTCATCATCTGCGGGTCGTTCTTCACGCTGAATCTGTTCATCGGTGTC

SH-12 ATTCGCGAGACCAACATCTACATGTACCTCTACTTTGTGTTCTTCATCATCTGCGGGTCGTTCTTCACGCTGAATCTGTTCATCGGTGTC

SH-16 ATTCGCGAGACCAACATCTACATGTACCTCTACTTTGTGTTCTTCATCATCTGCGGGTCGTTCTTCACGCTGAATCTGTTCATCGGTGTC

#FF **F1534F**

FJ-2 ATTCGCGAGACCAACATCTACATGTACCTCTACTTCGTGTTCTTCATCATCTTCGGGTCGTTCTTCACCCTCAATCTGTTCATCGGTGTC

FJ-3 ATTCGCGAGACCAACATCTACATGTACCTCTACTTCGTGTTCTTCATCATCTTCGGGTCGTTCTTCACCCTCAACCTGTTCATCGGTGTC

FJ-7 ATTCGCGAGACCAACATCTACATGTACCTCTACTTCGTGTTCTTCATCATCTTCGGGTCGTTCTTCACCCTCAATCTGTTCATCGGTGTC

FJ-8 ATTCGCGAGACCAACATCTACATGTACCTCTACTTCGTGTTCTTCATCATCTTCGGGTCGTTCTTCACCCTCAATCTGTTCATCGGTGTC

FJ-12 ATTCGCGAGACCAACATCTACATGTACCTCTACTTCGTGTTCTTCATCATCTTCGGGTCGTTCTTCACCCTCAATCTGTTCATCGGTGTC

FJ-16 ATTCGCGAGACCAACATCTACATGTACCTCTACTTCGTGTTCTTCATCATCTTCGGGTCGTTCTTCACCCTCAACCTGTTCATCGGTGTC

FJ-17 ATTCGCGAGACCAACATCTACATGTACCTCTACTTCGTGTTCTTCATCATCTTCGGGTCGTTCTTCACCCTCAACCTGTTCATCGGTGTC

FJ-25 ATTCGCGAGACCAACATCTACATGTACCTCTACTTCGTGTTCTTCATCATCTTCGGGTCGTTCTTCACCCTCAACCTGTTCATCGGTGTC

FJ-26 ATTCGCGAGACCAACATCTACATGTACCTCTACTTCGTGTTCTTCATCATCTTCGGGTCGTTCTTCACCCTCAACCTGTTCATCGGTGTC

FJ-28 ATTCGCGAGACCAACATCTACATGTACCTCTACTTCGTGTTCTTCATCATCTTCGGGTCGTTCTTCACCCTCAACCTGTTCATCGGTGTC

FJ-36 ATTCGCGAGACCAACATCTACATGTACCTCTACTTCGTGTTCTTCATCATCTTCGGGTCGTTCTTCACCCTCAACCTGTTCATCGGTGTC

FJ-48 ATTCGCGAGACCAACATCTACATGTACCTCTACTTCGTGTTCTTCATCATCTTCGGGTCGTTCTTCACCCTCAACCTGTTCATCGGTGTC

FJ-50 ATTCGCGAGACCAACATCTACATGTACCTCTATTTCGTGTTCTTCATCATCTTCGGGTCGTTCTTCACCCTCAACCTGTTCATCGGTGTC

FJ-51 ATTCGCGAGACCAACATCTACATGTACCTCTACTTCGTGTTCTTCATCATCTTCGGGTCGTTCTTCACCCTCAACCTGTTCATCGGTGTC

FJ-52 ATTCGCGAGACCAACATCTACATGTACCTCTATTTCGTGTTCTTCATCATCTTCGGGTCGTTCTTCACCCTCAACCTGTTCATCGGTGTC

FJ-56 ATTCGCGAGACCAACATCTACATGTACCTCTACTTCGTGTTCTTCATCATCTTCGGGTCGTTCTTCACTCTCAACCTGTTCATCGGTGTC

FJ-58 ATTCGCGAGACCAACATCTACATGTACCTCTACTTCGTGTTCTTCATCATCTTCGGGTCGTTCTTCACCCTCAATCTGTTCATCGGTGTC

FJ-61 ATTCGCGAGACCAACATCTACATGTACCTCTACTTCGTGTTCTTCATCATCTTCGGGTCGTTCTTCACCCTCAACCTGTTCATCGGTGTC

FJ-62 ATTCGCGAGACCAACATCTACATGTACCTCTATTTCGTGTTCTTCATCATCTTCGGGTCGTTCTTCACCCTCAACCTGTTCATCGGTGTC

FJ-63 ATCCGCGAGACCAACATCTACATGTACCTCTACTTTGTGTTCTTCATCATCTTCGGGTCGTTCTTCACCCTCAACCTGTTCATCGGTGTC

GZ-25 ATTCGCGAGACCAACATCTACATGTACCTCTACTTCGTGTTCTTCATCATCTTCGGGTCGTTCTTCACCCTCAACCTGTTCATCGGTGTC

NJ-3 ATTCGCGAGACCAACATCTACATGTACCTCTACTTCGTGTTCTTCACCATCTTCGGGTCGTTCTTCACCCTCAATCTGTTCATCGGTGTC

NJ-4 ATTCGCGAGACCAACATCTACATGTACCTCTACTTCGTGTTCTTCATCATCTTCGGGTCGTTCTTCACCCTCAACCTGTTCATCGGTGTC

NJ-5 ATTCGCGAGACCAACATCTACATGTACCTCTACTTCGTGTTCTTCATCATCTTCGGGTCGTTCTTCACCCTCAACCTGTTCATCGGTGTC

NJ-6 ATTCGCGAGACCAACATCTACATGTACCTCTACTTCGTGTTCTTCATCATCTTCGGGTCGTTCTTCACCCTCAACCTGTTCATCGGTGTC

NJ-7 ATTCGCGAGACCAACATCTACATGTACCTCTACTTCGTGTTCTTCATCATCTTCGGGTCGTTCTTCACCCTCAACCTGTTCATCGGTGTC

NJ-10 ATTCGCGAGACCAACATCTACATGTACCTCTACTTCGTGTTCTTCATCATCTTCGGGTCGTTCTTCACCCTCAACCTGTTCATCGGTGTC

NJ-13 ATTCGCGAGACCAACATCTACATGTACCTCTACTTCGTGTTCTTCATCATCTTCGGGTCGTTCTTCACCCTCAACCTGTTCATCGGTGTC

NJ-14 ATTCGCGAGACCAACATCTACATGTACCTCTACTTCGTGTTCTTCATCATCTTCGGGTCGTTCTTCACCCTCAACCTGTTCATCGGTGTC

NJ-17 ATTCGCGAGACCAACATCTACATGTACCTCTACTTCGTGTTCTTCATCATCTTCGGGTCGTTCTTCACCCTCAACCTGTTCATCGGTGTC

#FS **F1534S**

FJ-1 ATTCGCGAGACCAACATCTACATGTACCTCTACTTCGTGTTCTTCATCATCTTCGGGTCGTTCTTCACC

FJ-4 ATTCGCGAGACCAACATCTACATGTACCTCTACTTCGTGTTCTTCATCATCTTCGGGTCGTTCTTCACC

FJ-5 ATTCGCGAGACCAACATCTACATGTACCTCTACTTCGTGTTCTTCATCATCTTCGGGTCGTTCTTCACC

FJ-6 ATTCGCGAGACCAACATCTACATGTACCTCTACTTCGTGTTCTTCATCATCTTCGGGTCGTTCTTCACC

FJ-9 ATTCGCGAGACCAACATCTACATGTACCTCTACTTCGTGTTCTTCATCATCTTCGGGTCGTTCTTCACC

FJ-10 ATTCGCGAGACCAACATCTACATGTACCTCTACTTCGTGTTCTTCATCATCTTCGGGTCGTTCTTCACC

FJ-13 ATTCGCGAGACCAACATCTACATGTACCTCTACTTCGTGTTCTTCATCATCTTCGGGTCGTTCTTCACC

FJ-14 ATTCGCGAGACCAACATCTACATGTACCTCTACTTCGTGTTCTTCATCATCTTCGGGTCGTTCTTCACC

FJ-15 ATTCGCGAGACCAACATCTACATGTACCTCTACTTCGTGTTCTTCATCATCTTCGGGTCGTTCTTCACC

FJ-18 ATTCGCGAGACCAACATCTACATGTACCTCTACTTCGTGTTCTTCATCATCTTCGGGTCGTTCTTCACC

FJ-19 ATTCGCGAGACCAACATCTACATGTACCTCTACTTCGTGTTCTTCATCATCTCCGGGTCGTTCTTCACC

FJ-21 ATTCGCGAGACCAACATCTACATGTACCTCTACTTCGTGTTCTTCATCATCTTCGGGTCGTTCTTCACC

FJ-22 ATTCGCGAGACCAACATCTACATGTACCTCTACTTCGTGTTCTTCATCATCTTCGGGTCGTTCTTCACC

FJ-23 ATTCGCGAGACCAACATCTACATGTACCTCTACTTCGTGTTCTTCATCATCTTCGGGTCGTTCTTCACC

FJ-29 ATTCGCGAGACCAACATCTACATGTACCTCTACTTCGTGTTCTTCATCATCTTCGGGTCGTTCTTCACC

FJ-31 ATTCGCGAGACCAACATCTACATGTACCTCTACTTCGTGTTCTTCATCATCTTCGGGTCGTTCTTCACC

FJ-37 ATTCGCGAGACCAACATCTACATGTACCTCTACTTCGTGTTCTTCATCATCTTCGGGTCGTTCTTCACC

FJ-38 ATTCGCGAGACCAACATCTACATGTACCTCTACTTCGTGTTCTTCATCATCTTCGGGTCGTTCTTCACC

FJ-40 ATTCGCGAGACCAACATCTACATGTACCTCTACTTCGTGTTCTTCATCATCTTCGGGTCGTTCTTCACC

FJ-41 ATTCGCGAGACCAACATCTACATGTACCTCTACTTCGTGTTCTTCATCATCTTCGGGTCGTTCTTCACC

FJ-42 ATTCGCGAGACCAACATCTACATGTACCTCTACTTCGTGTTCTTCATCATCTTCGGGTCGTTCTTCACC

FJ-43 ATTCGCGAGACCAACATCTACATGTACCTCTACTTCGTGTTCTTCATCATCTTCGGGTCGTTCTTCACC

FJ-46 ATTCGCGAGACCAACATCTACATGTACCTCTACTTCGTGTTCTTCATCATCTTCGGGTCGTTCTTCACC

FJ-49 ATTCGCGAGACCAACATCTACATGTACCTCTATTTCGTGTTCTTCATCATCTTCGGGTCGTTCTTCACC

FJ-53 ATTCGCGAGACCAACATCTACATGTACCTCTACTTCGTGTTCTTCATCATCTTCGGGTCGTTCTTCACC

FJ-54 ATTCGCGAGACCAACATCTACATGTACCTCTACTTCGTGTTCTTCATCATCTTCGGGTCGTTCTTCACC

FJ-55 ATTCGCGAGACCAACATCTACATGTACCTCTACTTCGTGTTCTTCATCATCTTCGGGTCGTTCTTCACC

FJ-57 ATTCGCGAGACCAACATCTACATGTACCTCTATTTCGTGTTCTTCATCATCTTCGGGTCGTTCTTCACC

FJ-60 ATTCGCGAGACCAACATCTACATGTACCTCTATTTCGTGTTCTTCATCATCTTCGGGTCGTTCTTCACC

FJ-64 ATTCGCGAGACCAACATCTACATGTACCTCTATTTCGTGTTCTTCATCATCTTCGGGTCGTTCTTCACC

GZ-1 ATTCGCGAGACCAACATCTACATGTACCTCTACTTCGTGTTCTTCATCATCTTCGGGTCGTTCTTCACC

GZ-3 ATTCGCGAGACCAACATCTACATGTACCTCTACTTCGTGTTCTTCATCATCTCCGGGTCGTTCTTCACC

GZ-6 ATTCGCGAGACCAACATCTACATGTACCTCTACTTCGTGTTCTTCATCATCTTCGGGTCGTTCTTCACC

GZ-10 ATTCGCGAGACCAACATCTACATGTACCTCTACTTCGTGTTCTTCATCATCTCCGGGTCGTTCTTCACC

GZ-12 ATTCGCGAGACCAACATCTACATGTACCTCTACTTCGTGTTCTTCATCATCTTCGGGTCGTTCTTCACC

GZ-16 ATTCGCGAGACCAACATCTACATGTACCTCTACTTCGTGTTCTTCATCATCTTCGGGTCGTTCTTCACC

GZ-18 ATTCGCGAGACCAACATCTACATGTACCTCTACTTCGTGTTCTTCATCATCTCCGGGTCGTTCTTCACC

GZ-19 ATTCGCGAGACCAACATCTACATGTACCTCTACTTCGTGTTCTTCATCATCTCCGGGTCGTTCTTCACC

GZ-23 ATTCGCGAGACCAACATCTACATGTACCTCTACTTCGTGTTCTTCATCATCTCCGGGTCGTTCTTCACC

GZ-24 ATTCGCGAGACCAACATCTACATGTACCTCTACTTCGTGTTCTTCATCATCTCCGGGTCGTTCTTCACC

GZ-28 ATTCGCGAGACCAACATCTACATGTACCTCTACTTCGTGTTCTTCATCATCTCCGGGTCGTTCTTCACC

GZ-30 ATTCGCGAGACCAACATCTACAGGTACCTCTACTTCGTGTTCTTCATCATCTCCGGGTCGTTCTTCACC

GZ-32 ATTCGCGAGACCAACATCTACATGTACCTCTACTTCGTGTTCTTCATCATCTCCGGGTCGTTCTTCACC

#SC **S1534C**

XZ-19 ATTCGCGAGACCAACATCTACATGTACCTCTACTTCGTGTTCTTCATCATCTCCGGGTCGTTCTTCACCCTCAACCTGTTCATCGGTGTC

FJ-27 ATTCGCGAGACCAACATCTACATGTACCTCTACTTCGTGTTCTTCATCATCTGCGGGTCGTTCTTCACCCTCAATCTGTTCATCGGTGTC

FJ-44 ATTCGCGAGACCAACATCTACATGTACCTCTACTTCGTGTTCTTCATCATCTGCGGGTCGTTCTTCACCCTCAATCTGTTCATCGGTGTC

TJ-37 ATTCGCGAGACCAACATCTACATGTACCTCTACTTCGTGTTCTTCATCATCTCCGGGTCGTTCTTCACCCTCAATCTGTTCATCGGTGTC

TJ-39 ATTCGCGAGACCAACATCTACATGTACCTCTACTTCGTGTTCTTCATCATCTCCGGGTCGTTCTTCACCCTCAATCTGTTCATCGGTGTC

TJ-43 ATTCGCGAGACCAACATCTACATGTACCTCTACTTCGTGTTCTTCATCATCTGCGGGTCGTTCTTCACCCTCAATCTGTTCATCGGTGTC

TJ-48 ATTCGCGAGACCAACATCTACATGTACCTCTACTTCGTGTTCTTCATCATCTCCGGGTCGTTCTTCACCCTCAATCTGTTCATCGGTGTC

BYS12 ATTCGCGAGACCAACATCTACATGTACCTCTACTTCGTGTTCTTCATCATCTGCGGGTCGTTCTTCACCCTCAACCTGTTCATCGGTGTC

BYS28 ATTCGCGAGACCAACATCTACATGTACCTCTACTTTGTGTTCTTCATCATCTCCGGGTCGTTCTTCACCCTCAACCTGTTCATCGGTGTC

YX-54 ATTCGCGAGACCAACATCTACATGTACCTCTACTTCGTGTTCTTCATCATCTCCGGGTCGTTCTTCACCCTCAATCTGTTCATCGGTGTC

XZ-6 ATTCGCGAGACCAACATCTACATGTACCTCTACTTCGTGTTCTTCATCATCTGCGGGTCGTTCTTCACCCTCAATCTGTTCATCGGTGTC

XZ-12 ATTCGCGAGACCAACATCTACATGTACCTCTACTTCGTGTTCTTCATCATCTCCGGGTCGTTCTTCACCCTCAATCTGTTCATCGGTGTC

XZ-32 ATTCGCGAGACCAACATCTACATGTACCTCTACTTCGTGTTCTTCATCATCTCCGGGTCGTTCTTCACCCTCAATCTGTTCATCGGTGTC

#FL  **F1534L**

BY-4 ATTCGCGAGACCAACATCTACATGTACCTCTACTTCGTGTTCTTCATCATCTTCGGGTCGTTCTTCACCCTCAACCTGTTCATCGGTGTC

BY-16 ATTCGCGAGACCAACATCTACATGTACCTCTACTTCGTGTTCTTCATCATCTTCGGGTCGTTCTTCACCCTCAATCTGTTCATCGGTGTC

HP-17 ATTCGCGAGACCAACATCTACATGTACCTCTACTTCGTGTTCTTCATCATCTTGGGGTCGTTCTTCACCCTCAACCTGTTCATCGGTGTC

BYS18 ATTCGCGAGACCAACATCTACATGTACCTCTACTTCGTGTTCTTCATCATCCTCGGGTCGTTCTTCACCCTCAACCTGTTCATCGGTGTC

BYS20 ATTCGCGAGACCAACATCTACATGTACCTCTACTTCGTGTTCTTCATCATCTTGGGGTCGTTCTTCACCCTCAACCTGTTCATCGGTGTC

JH-32 ATTCGCGAGACCAACATCTACATGTACCTCTACTTCGTGTTCTTCATCATCTTGGGGTCGTTCTTCACCCTCAATCTGTTCATCGGTGTC

BYS-3 ATTCGCGAGACCAACATCTACATGTACCTCTACTTCGTGTTCTTCATCATCTTGGGGTCGTTCTTCACCCTCAACCTGTTCATCGGTGTC

YX-4 ATTCGCGAGACCAACATCTACATGTACCTCTACTTCGTGTTCTTCATCATCTTGGGGTCGTTCTTCACCCTCAACCTGTTCATCGGTGTC

#LL **L1534L**

HP-6 ATTCGCGAGACCAACATCTACATGTACCTCTACTTCGTGTTCTTCATCATCTTGGGGTCGTTCTTCACCCTCAACCTGTTCATCGGTGTC
